# Bovine rumen impaction caused by ingestion of *Gonometa postica* cocoons in eastern-central Namibia

**DOI:** 10.4102/jsava.v90i0.1995

**Published:** 2019-10-28

**Authors:** Alaster Samkange, Magrecia Hausiku, Erick Kandiwa, Pricilla Mbiri, Erdwin N. Muradzikwa, Borden Mushonga

**Affiliations:** 1 School of Veterinary Medicine, University of Namibia, Windhoek, Namibia

**Keywords:** *Gonometa postica*, Cocoons, Rumen Impaction, Bovine, Namibia

## Abstract

Cases of rumen impaction caused by ingestion of *Gonometa postica* cocoons occurred at a farm in eastern-central Namibia. Ten animals died on the farm over the previous 5 months. Rumenotomies were successfully performed on three affected animals. The authors were of the opinion that the affected animals ingested the cocoons around the time of weaning, which then resulted in tangled masses that gradually stretched and enlarged because of entrapment of ingesta, eventually causing impaction of the rumen in the young adult animals. These are the first reported cases of ruminal impaction attributable to *G. postica* cocoon ingestion in Namibia.

## Introduction

Rumen impaction occurs when there is an accumulation of indigestible materials in the rumen that impedes the normal flow of ingesta, causing over-filling of the rumen, which leads to abdominal distension and discomfort, inappetence, poor degradation and fermentation of rumen contents, culminating in the production of scanty or no faeces and even death (Alimi et al. 2018). These indigestible materials may include foreign bodies such as plastic bags, nylon, rope, metallic nails, wires, stones, fruit seeds, hairballs, leather materials, clothing materials (Bwatota, Makungu & Nonga 2018; Mushonga et al. 2015; Tesfaye & Chanie 2012; Vanitha et al. 2010) and even plant material (Zhai et al. 2013). Ingestion of *Gonometa postica* (better known as Molopo, burn worm or *brandwurm*) (Zumpt 1971) and *Gonometa rufobrunnea* (Edwards 1935) cocoons have also been reportedly responsible for the outbreaks of ruminal impaction in cattle, resulting in high mortality rates. Rumen impaction is therefore economically very important because it results in poor productivity and high mortality rates (Bwatota et al. 2018; Mushonga et al. 2015).

*Gonometa* species occur abundantly in southern Africa, specifically in the North West and Northern Cape provinces of South Africa, and in Botswana as well as Namibia (Veldtman, McGeoch & Scholtz 2004). According to Von Seydlitz (1999), even though *Gonometa* species cause deaths in cattle because of rumen impaction in Namibia, they also present a business opportunity for the silk manufacturing industry in the country (Bause 2005).

This report describes cases of rumen impaction and deaths in cattle caused by the ingestion of *G. postica* cocoons that occurred at a farm in the Omaheke region of Namibia. Although the local communities and the veterinary fraternity in Namibia have been aware of this condition for decades, reports of such incidences have only appeared in grey literature (newspapers, magazines and annual reports). This case report is, to the best of our knowledge, the first documented, peer-reviewed, scientific publication of rumen impaction in cattle caused by *G. postica* cocoon ingestion in Namibia.

## Case report

A farmer from the Omaheke region in eastern-central Namibia reported, to the ambulatory clinic service of the University of Namibia’s School of Veterinary Medicine, that many of his animals had distended abdomens. Eleven animals out of a herd of 78 cattle had reportedly died of a similar condition over a 5-month period (October 2018–February 2019). The farmer had consistently observed large amounts of impacted rumen contents in the animals that had died. He had never experienced this particular problem on the farm prior to October 2018, but neighbouring farms had reportedly experienced similar problems.

**FIGURE 1:**
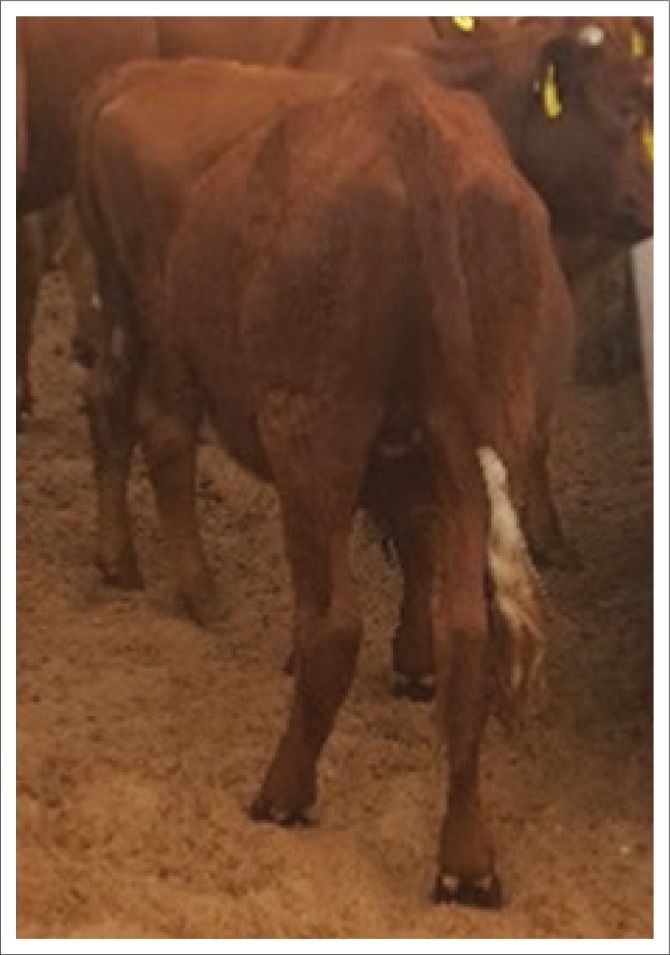
A steer showing typing ruminal distension because of impaction caused by the ingestion of *Gonometa postica* cocoons.

Upon arrival at the farm, about 10% (*n* = 78) of the cattle had various degrees of abdominal distension, loss of condition and long, dull, loose hair coats (Figure 1). Younger animals aged 12–24 months old (*n* = 7) were more affected than older animals, and both sexes were affected to the same degree. The pastures were severely overgrazed, although there were numerous acacia shrubs and trees with green leaves and pods. Also noticeable were numerous *G. postica* cocoons on acacia trees (up to 23 cocoons per tree), with evidence of recent browsing wherever trees were accessible to cattle, especially around or near the calf sheds.

Three severely affected animals from the herd were selected for closer clinical examination. The rectal temperatures were normal, but the pulse and respiration rates were moderately elevated, and the capillary refill times were less than 2 seconds. The rumens were grossly distended with a doughy consistency on palpation and also showing slight bulging on the left side (Figure 1). Weak and incomplete ruminal contractions occurred at not more than one contraction every 3 minutes. This clinical picture was consistent in all three animals examined. A tentative diagnosis of rumen impaction because of ingestion of Molopo worm cocoons was arrived at based on the history, clinical presentation, epidemiology and the presence of numerous cocoons on the farm. Exploratory rumenotomies were then performed on the three animals.

**FIGURE 2:**
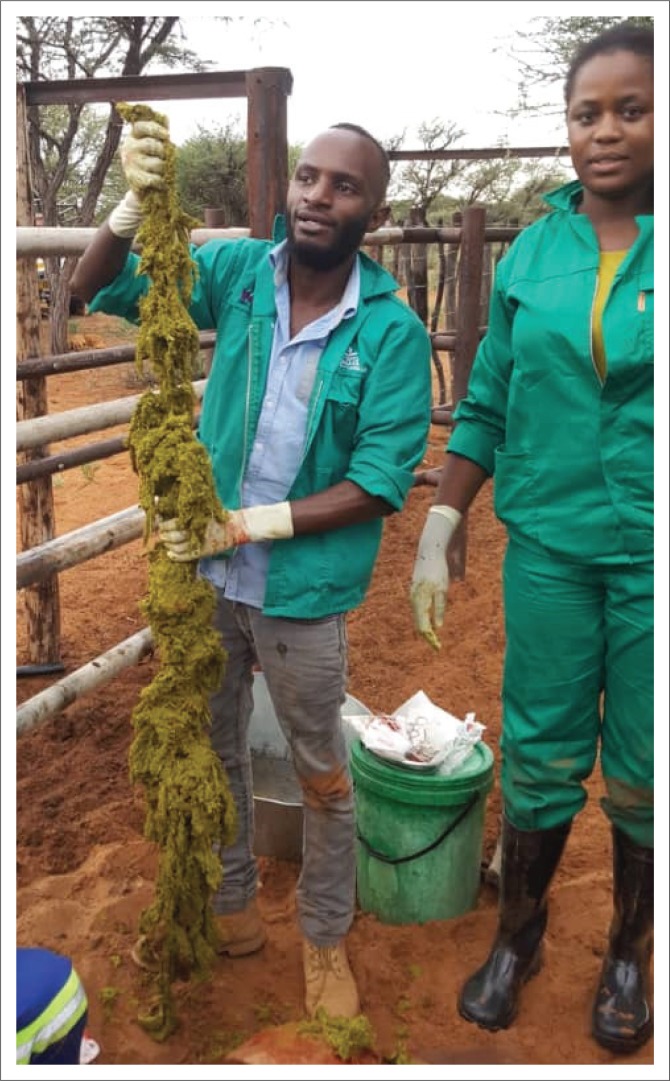
Impacted material retrieved from the steer during rumenotomy.

Each patient was aseptically prepared for the rumenotomy procedure. Large masses of impacted materials were discovered during exploratory rumenotomies on all three animals, and these were removed (Figure 2). The impacted ingesta comprised strong silk strands and rumen content, a typical finding in cases of *G. postica* cocoon impaction. All three animals recovered well from the surgery and showed a marked improvement in their general habitus and appetite on subsequent visits to the farm.

## Ethical considerations

The surgeries were performed using standard protocols and the animals were handled humanely and in strict adherence to Namibia’s animal welfare guidelines.

## Discussion

**FIGURE 3:**
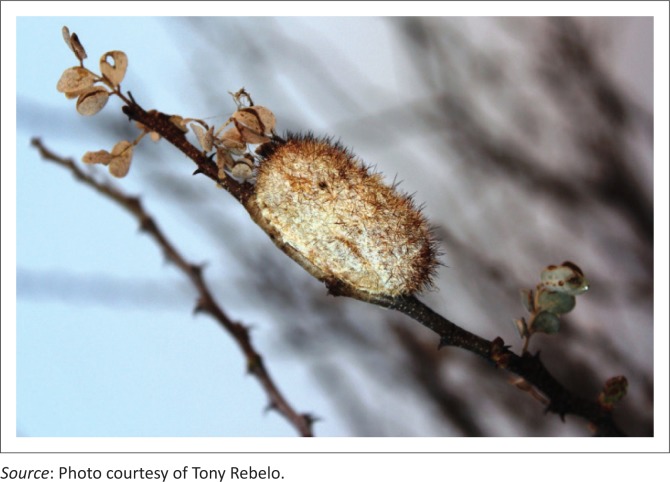
*Gonometa postica* cocoon with characteristic spikes clearly visible.

**FIGURE 4:**
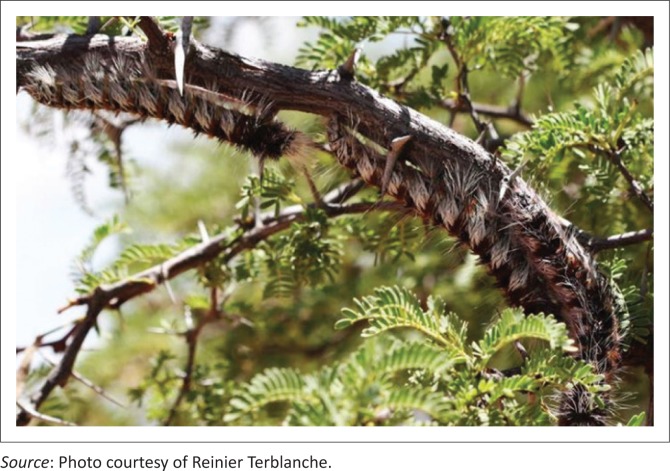
African Wild Silk Moth, *Gonometa postica*, caterpillars resting on *Vachellia erioloba* (Camel Thorn) branches, Eastern Kalahari Bioregion.

In southern Africa only two indigenous moth species, *G. postica* (Figure 3 and 4) and *G. rufobrunnea* Aurivillius (Lasiocampidae), produce high-quality commercial silk (Veldtman, McGeoch & Scholtz 2002; Veldtmann et al. 2004). The fibroin derived from both cocoons has been found to exhibit very similar chemical, structural and thermal properties (Mhuka, Dube & Nindi 2013). Unfortunately, when ingested by ruminants, these cocoons (Figure 3) are indigestible and thus accumulate in the rumen, resulting in rumen impaction and inevitable death from starvation (Edwards 1935; Zumpt 1971). *Gonometa postica*, also commonly known as the Molopo worm or African silk moth, is the only commercial-quality silk-producing moth species found in Namibia (Veldtman et al. 2002).

Although *G. postica* is polyphagous, with host trees that include *Vachellia erioloba* (formerly *Acacia erioloba*) (camelthorn) (Figure 4), *Vachellia tortilis* (formerly *Acacia tortilis*) (umbrella thorn acacia), *Senegalia mellifera* (formerly *Acacia mellifera*) (blackthorn), *Burkea africana* (wild syringa), *Brachystegia* spp. (miombo) and the exotic tree *Prosopis glandulosa* (honey mesquite) (Veldtman et al. 2002), the Namibian Molopo worms are predominantly found on camelthorn and blackthorn trees (Von Seydlitz 1999). The high incidence of ruminal impactions attributable to cocoon ingestion is thus not surprising in the acacia veld, which is predominant in the dry eastern-central parts of Namibia (Von Seydlitz 1999). Indeed, these cocoons have long been blamed by local communities to be the cause of deaths of cattle, antelope and other ruminants in the Kalahari. Some Zimbabwean communities have, for many years, also blamed the cocoons for deaths of both commercial and communal livestock.

Two theories of *G. postica* ingestions have since arisen from previous reports. One theory states that ingestion occurs during drought years when adult cattle ingest *G. postica* cocoons because they resemble acacia pods on acacia trees (Bafana 2009). Following ingestion of the *G. postica* cocoons, the indigestible silk accumulates in the rumen with the watery environment causing the fibres to swell without breaking up. This results in entrapment of the rumen contents between the silk strands, thereby forming a solid indigestible mass. The tangled silk strands progressively stretch and build up because of the gradual addition of more ingesta, thus enlarging the mass (Figure 2), culminating in the mechanical blockage of the rumen, accompanied by impaired primary and secondary rumen contractions. Eventually this leads to progressive loss of condition, weakness and death of the animal from starvation. The drought of the 2018 and 2019 rainy season in Namibia might have resulted in more frequent ingestion of *G. pastica* cocoons by cattle, leading to widespread incidents of cattle dying from rumen impaction. This scenario, however, would not have precipitated the problem in one season so soon after the drought. According to the literature, the rumen impaction because of *G. postica* ingestion is a slow progressive problem.

The second theory states that calves ingest Molopo worm cocoons during the time of weaning. Zumpt (1971) reported rumen impaction in animals that were otherwise adequately fed. He proposed that *G. postica* cocoon ingestion occurred when the cocoons fell from acacia trees that are left to grow and provide shade around calf paddocks and that calves ingested the cocoons as a result of either the pica, inquisitiveness or hunger of naive calves at the time of weaning. The silk in the cocoon then progressively accumulates, matting with and trapping food particles and enlarging in the process. By the time the calves become young adults, the impacted mass would have become large and would have begun to interfere with rumen contractions, often causing a noticeable potbelly appearance in the affected animals (Figure 2). Although both theories can be sustained by the circumstances of the present report, the second one is more appealing to the authors. The finding of rumen distention in young animals in this study, therefore, comes as no surprise.

*In vitro* tests on the Molopo worm cocoon fibres have determined that substances like concentrated sulphuric acid, nitric acid and acetic acid have no effect on these silk fibres (Zumpt 1971). Medical treatment of the impaction caused by these cocoons is therefore futile. Rumenotomy and physical removal of the impacted material is the only viable treatment option (Edwards 1935; Zumpt 1971).

In southern African cultures and in other similar cultures around the world, the cocoons are used to make traditional items like ankle rattles, hand rattles, necklaces, purses or other artefacts (Peigler 2019). In countries like Namibia, Botswana, Kenya and South Africa as well as Madagascar (where the silk is used ritually for wrapping of the dead), the cocoons are harvested commercially, with the latter country having done so for centuries already (Torbitt 2006). This information, therefore, presents a unique opportunity for the prevention of cocoon impaction in livestock through commercial exploitation of these cocoons.

Following awareness campaigns in areas where the cocoons present as an existing problem, affected and sensitised communities should be encouraged to harvest the cocoons as a commercial income-generating enterprise targeting local wild silk companies. In this way, a win–win and sustainable development synergy scenario is potentially created between farmers and the silk industry. Although such projects have previously been initiated in nearby Leonardville, in the Omaheke region (Torbitt 2006; Von Seydlitz 1999) and Zimbabwe (Bafana 2009) in the past, more emphasis needs to be placed on awareness campaigns in potentially affected areas.
